# Modeling growth of *Alicyclobacillus acidoterrestris* DSM 3922 type strain vegetative cells in the apple juice with nisin and lysozyme

**DOI:** 10.3934/microbiol.2017.2.315

**Published:** 2017-05-05

**Authors:** Celenk Molva, Ayse Handan Baysal

**Affiliations:** Department of Food Engineering, Izmir Institute of Technology, Urla, 35430, Izmir, Turkey

**Keywords:** *Alicyclobacillus* acidoterrestris, apple juice, growth, natural antimicrobials, modeling

## Abstract

In the present study, the effect of storage temperature on *A. acidoterrestris* DSM 3922 cells (10^5^ CFU/mL) was examined during growth in reconstituted apple juice (pH 3.8, °Brix 11.3) containing nisin (0–100 IU/mL) and lysozyme (0–100 mg/L). The growth curves were obtained at three temperatures of 27, 35 and 43 °C using absorbance data (OD_600 nm_). Based on the results, the minimal inhibitory concentrations (MICs) of nisin were found as 10 IU/mL at all tested temperatures. On the other hand, increasing the temperature decreased the amount of lysozyme for growth inhibition. The MICs of lysozyme were found as 10, 2.5 and 1.25 mg/L at 27, 35 and 43 °C, respectively. At selected non-inhibitory doses, nisin (1.25–5 IU/mL) and lysozyme (0.3–2.5 mg/L) prolonged the lag time compared to the controls at the corresponding temperatures. In addition, there was a strong linear relationship between the lag time and lysozyme concentrations at 27 and 35 °C (R^2^ > 0.98). The results of this study demonstrated that both nisin and lysozyme could be used to inhibit the growth of *A. acidoterrestris* cells in the apple juice. The results also indicated that the growth parameters were variable depending on the storage temperature and the type of the antimicrobial agent used in the apple juice.

## 1. Introduction

*Alicyclobacillus acidoterrestris* is a thermoacidophilic, non-pathogenic, rod-shaped, spore-forming bacterium. It has the ability to grow in a wide temperature (25–60 °C) and pH (2.5–6.0) ranges [1]. ω-alicyclic fatty acids are the major lipid components and provide its resistance to acidic conditions and high temperatures [2]. After fruit juice pasteurization, its spores can germinate into vegetative cells leading to spoilage [3]. Spoilage is difficult to detect visually [4] and has been detected in various pasteurized juice samples such as apple, tomato, white grape, grapefruit, orange, and pineapple juices but not in red grape juices and Cupuaçu [1].

The use of natural antimicrobials in foods inhibits the growth of many microorganisms without health risks to consumers [5]. In the food industry, it is important to determine the effects of non-thermal technologies on microbial inactivation and the growth parameters of the survivors such as the lag phase (λ) and the maximum specific growth rate (µ) [6]. Nisin is a heat stable bacteriocin produced by *Lactococcus* *lactis* sub sp. *lactis*. It shows antibacterial activity against Gram-positive bacteria and spore formers such as *Bacillus* and *Clostridium* spp. This antibacterial agent is essentially non-toxic to humans and degraded without causing any damage to the intestinal tract. In the United States, it was approved by the Food and Drug Administration (FDA) and allowed to be used in processed cheeses [7]. In the food industry, it has been used as a natural preservative in processed cheese, milk, dairy products, canned foods, hot baked flour products and pasteurized liquid egg [8]. On the other hand, its use is limited to 15 ppm in meat products by the FDA [9]. Nisin could be added directly to the juices [10],[11],[12] or incorporated in polymers for controlled release during storage against *A. acidoterrestris* [13]. Similar to nisin, hen egg white lysozyme has Generally Recognized as Safe (GRAS) status. It has been used as a food preservative in cheeses, cow's milk, beer, fresh fruits and vegetables, fish, meat and wine [8]. Also, lysozyme could inhibit the growth of *A. acidoterrestris* in solution, incorporated in water-soluble polyvinyl alcohol films or immobilized on polymer films [14]. Due to its safety and high technological stability, it is considered as an ideal food preservative. European Union allergen legistration requires its labeling because of its ability to cause allergies [15].

Temperature is one of the important extrinsic factors that affect the microbial growth and can be easily changed during food processing and storage. Many predictive models have been developed to investigate the growth kinetics under different temperature conditions [16]. To our knowledge, there is no study about the effect of storage temperature on the antimicrobial activities of nisin and lysozyme against *A. acidoterrestris* in juice. Therefore, the objectives of the present study were to determine the inhibitory effects of nisin and lysozyme against *A. acidoterrestris* DSM 3922 cells in the apple juice at different storage temperatures (27, 35 and 43 °C) and finally to estimate the growth kinetic parameters at non-inhibitory doses.

## 2. Materials and Method

### 2.1. Bacterial strain

*A. acidoterrestris* DSM 3922 type strain was kindly provided by Karl Poralla (Deutsche SammLung von Mikroorganismen und Zellkulturen's collection, Braunschweig, Germany). This culture was grown on *Bacillus acidoterrestris* Agar (pH 4.0, Merck, Germany) for 2 days at 43 °C, and then stored in 20% glycerol-BAT broth (Döhler, Germany) at –80 °C for further analysis.

### 2.2. Juice samples

Concentrated apple juice (70.3° Brix) was kindly provided by ASYA Fruit Juice and Food Ind. Inc. (Isparta, Turkey) and reconstituted to 11.30 °Brix by a refractometer (Mettler Toledo, USA). The presence of *Alicyclobacillus* spp. contamination in juice samples was determined by membrane filtration method as previously described [17]. The pH of the juice was measured as 3.82 ± 0.01 (Hanna instruments, Hungary).

### 2.3. Antimicrobials

Lysozyme (≥40,000 units/mg protein) from hen egg white and nisin from *Lactococcus lactis* (2.5%, balance sodium chloride and denatured milk solids) were obtained from Sigma Chem. Co. (St Louis, MO, USA).

### 2.4. Growth study and modeling

First, *A. acidoterrestris* cells were grown overnight on Potato dextrose agar (Difco, BD) at 43 °C. The colonies were suspended in 10 mL Maximum Recovery Diluent (Oxoid) to obtain a bacterial density of McFarland 1.0 (10^6^ CFU/mL) using a densitometer (Den-1, HVD Life Sciences, Austria). After centrifugation at 16,000 × g for 5 min, the pellet was dissolved in 10 mL apple juice. Then, 180 µL juice containing antimicrobial (nisin 0–100 IU/mL or lysozyme, 0–100 mg/L) and 20 µL of the bacterial suspension were added into the wells of a flat bottom 96-well plate in duplicates (Corning Costar) for each temperature tested. The plates were incubated individually at 27, 35 or 43 °C in a microplate reader (Varioskan® Flash, Thermo, Finland). Inoculated apple juice without antimicrobials was used as control. Absorbance of the well contents was determined at 600 nm. The averages of absorbance values versus time were plotted to form growth curves. The MIC was defined as the lowest concentration required for the inhibition. Experimental growth data were fitted to the Baranyi growth model [18] in DMFit Version 2.1 software (Institute of Food Research, Norwick, UK) to estimate the growth parameters at non-inhibitory doses.

### 2.5. Model evaluation

The mean values and standard deviations were calculated by Excel (Microsoft Corp., USA). The fitting of the model to the experimental data was determined by coefficient of determination (R^2^) calculated by the DMFit 2.1 program. The higher R^2^ values provide better prediction by the fitted model [18].

## 3. Results and Discussion

### 3.1. MIC values of nisin and lysozyme in the apple juice

Growth inhibition of *A. acidoterrestris* in apple juice with nisin was independent on storage temperature. MICs were found as 10 IU/mL nisin under all conditions. Since nisin has great stability at high temperatures and low pH, especially at the range of pH 3–4, it has been used to inhibit or control the growth of *A. acidoterrestris* in highly acidic drinks [11]. The antimicrobial activity of nisin depends on food environment factors such as pH, lipid content, particle size, storage time, and structural uniformity [19]. During storage, antibacterial activity of nisin is increased in high pH foods and at high temperatures [20]. In the related literature, different MIC values have been reported for *Alicyclobacillus* spp. This may be due to nisin concentration, the characteristics of juice or medium and the tested species [21]. The MICs of nisin were also found lower than those of other studies but it is difficult to compare the obtained results with previous studies because of the use of different organisms, growth and incubation conditions [21].

In another study, Yamazaki et al. (2000) concluded that nisin has inhibitory effect in unclear commercial juices and no effect in clear drinks. They suggested that nisin may bind polyphenols present in unclear juices and growth may be inhibited because of the binding of nisin to some particles present in the unclear apple juice and the synergism between the polyphenols and nisin. In a previous study, cell growth was completely inhibited in the presence of 100 IU/mL nisin in grapefruit, apple and orange juices at 43 °C [10]. According to Yamazaki et al. (2000), cell growth was inhibited on agar plates by 1.56 to 25 IU/mL and 25 to 100 IU/mL at pH 3.4 and 4.2, respectively during incubation at 46 °C.

Storage at lower temperatures caused the use of higher amounts of lysozyme. The activity of lysozyme was increased at higher temperatures. Denatured lysozyme by heating was found to indicate enhanced antimicrobial activity due to the polymerization [22]. In this study, increasing the storage temperature may have improved the enzymatic activity of lysozyme against *A. acidoterrestris* by lowering the amount needed for inhibition. The MICs were 10, 2.5 and 1.25 mg/L at 27, 35 and 43 °C, respectively. In the related literature, it was found to exhibit the highest antimicrobial activity against *A. acidoterrestris* in saline solutions and the addition of lysozyme in laboratory medium resulted in a protective effect [23]. These researchers also found that the MICs of lysozyme towards vegetative cells ranged from 0.1–6 mg/L in acidified malt extract broth. We also found the MICs of lysozyme in the apple juice within this range at 35 °C and 43 °C.

### 3.2. Growth parameters after exposure to nisin and lysozyme in the apple juice

Although the measurement of the absorbance is useful to estimate its growth, it might help to compare the growth rate and lag time under different conditions [24]. Since growth models developed in sterile broth do not always provide reliable predictions of pathogen growth on non-sterile and non-homogenous foods [25], growth studies were achieved in apple juice instead of laboratory medium or model juice to estimate the growth parameters in this study. As seen from Tables 1 and 2, the growth curves fitted to the Baranyi model had high R^2^ values (≥0.98). The R^2^ values indicated that fitted models provided good fit to the experimental data.

**Table 1.** Estimated growth kinetic parameters of *A. acidoterrestris* cells in the apple juice with nisin at 27, 35 and 43 °C.

T (°C)	IU/mL	λ (h)	µ (h^−1^ 10^−4^)	R^2^
27	1.25	25.79 ± 0.56	16.98 ± 0.37	0.997
	2.5	29.83 ± 3.45	14.13 ± 1.67	0.997
	5.0	48.00 ± 1.69	18.07 ± 2.59	0.990
	Control	22.67 ± 0.59	10.13 ± 0.21	0.999
				
35	1.25	28.06 ± 0.62	33.40 ± 0.47	0.997
	2.5	31.42 ± 0.25	34.11 ± 1.09	0.998
	5.0	36.50 ± 0.79	36.90 ± 0.00	0.995
	Control	9.91 ± 1.80	28.58 ± 2.05	0.996
				
43	1.25	18.48 ± 0.01	51.37 ± 0.00	0.983
	2.5	10.1 ± 0.01	37.9 ± 0.03	0.992
	5.0	18.5 ± 0.00	51.4 ± 1.00	0.980
	Control	5.33 ± 0.04	33.76 ± 0.37	0.990

Values are estimated using an average of replicates obtained from growth curves. λ is lag phase (h); µ is specific growth rate (h^−1^ 10^−4^).

The estimated lag times decreased and growth rates increased with increasing storage temperature among the controls. The growth was favored at 43 °C with the highest growth rate and shortest lag times most probably due to the thermophilic nature of the tested organism. As seen from Table 1, there was a strong correlation between the growth rates and nisin concentrations at 35 °C (R^2^ > 0.91). In general, it is accepted that µ is not influenced by sublethal injuries after conventional preservation treatments and is identical to that of untreated cells. After exposure to some novel technologies such as pulsed electric fields and electron beam irradiation, the growth rate of surviving cells can be changed [6]. In the present study, the growth rates of the treated cells were higher than that of the controls within each temperature (Table 1). The addition of nisin in the apple juice resulted in an extension of lag phase compared to untreated control cells. We observed a relationship between the lag time and nisin concentrations at 27 and 35 °C (R^2^ > 0.83). At the highest non-inhibitory nisin dose, the increase in the lag time was approximately 25, 27 and 13 h at 27 °C, 35 °C and 43 °C, respectively.

The growth rates in the presence of lysozyme ranged from approximately 13.5 to 16.5, 22.2 to 31.8, and 31.6 to 36.6 × 10^−4^ h^−1^ at 27, 35, and 43 °C, respectively (Table 2). We observed a linear relationship between the rate and lysozyme concentration at 27 °C (R^2^ > 0.94). As the temperature decreased, the effect of lysozyme on the lag phase extension was more pronounced. There was a strong linear relationship between the lag time and lysozyme concentrations at 27 and 35 °C (R^2^ > 0.98). The treatment with 1.25, 2.5 and 5 mg/L lysozyme delayed the lag time approximately 8, 20 and 28 h at 27 °C, respectively. When the temperature was increased to 43 °C, the lag time was delayed with approximately 2, 2.5 and 9 h in the apple juice with 0.08, 0.16 and 0.3 mg/mL lysozyme, respectively. To our knowledge, there is only one study about the modeling growth kinetics of *A. acidoterrestris* in the broth with monolaurin using absorbance data [26]. They found that the Gompertz and Baranyi models were satisfactorily described the growth curves of cells in the presence of monolaurin. According to their results, monolaurin could be used to inhibit the growth and it was effective on cells by delaying the lag phase approximately 8 h and reducing the growth index from 48% to 32–37%.

**Table 2.** Estimated growth kinetic parameters of *A. acidoterrestris* cells in the apple juice with lysozyme at 27, 35 and 43 °C.

T (°C)	mg/L	λ (h)	µ (h^−1^ 10^−4^)	R^2^
27	1.25	30.53 ± 1.87	13.51 ± 0.27	0.999
	2.5	43.04 ± 1.46	14.41 ± 0.47	0.999
	5.0	51.10 ± 1.33	16.52 ± 0.30	0.994
	Control	22.67 ± 0.59	10.13 ± 0.21	0.999
				
35	0.3	16.69 ± 0.22	22.17 ± 0.04	0.989
	0.6	21.11 ± 0.31	25.28 ± 0.05	0.990
	1.25	25.71 ± 1.00	31.81 ± 0.40	0.986
	Control	9.91 ± 1.80	28.58 ± 2.05	0.996
				
43	0.08	7.06 ± 0.00	31.94 ± 0.00	0.991
	0.16	7.80 ± 0.04	31.56 ± 1.01	0.992
	0.3	14.71 ± 0.03	36.61 ± 0.90	0.996
	Control	5.33 ± 0.04	33.76 ± 0.37	0.990

Values are estimated using an average of replicates obtained from growth curves. λ is lag phase (h); µ is specific growth rate (h^−1^ 10^−4^).

## 4. Conclusion

We observed a significant difference in the amounts of lysozyme for growth inhibition at three temperatures tested. Both nisin and lysozyme were effective to delay lag time and increase growth rate when used at lower amounts. Differences in the antimicrobial action of nisin and lysozyme against *A. acidoterrestris* could be explained by variations in storage temperature. The obtained results from this study will be useful to inhibit or delay the growth of *A. acidoterrestris* by combining extrinsic factors such as storage temperature and natural antimicrobials as a hurdle concept to prolong the shelf-life of commercial apple juices.
